# The Relationship of 5-Aminolevulinic Acid on Mood and Coping Ability in Prediabetic Middle Aged and Older Adults

**DOI:** 10.3390/geriatrics3020017

**Published:** 2018-04-04

**Authors:** Rachael K. Aquino, Michael Perez, Payel Sil, Terry Shintani, Rosanne Harrigan, Beatriz Rodriguez

**Affiliations:** 1 Department of Geriatrics and Department of Complementary and Alternative Medicine, John A. Burns School of Medicine, University of Hawaii, Honolulu, HI 96813, USA; mhperez@hawaii.edu (M.P.); terrys@hawaii.edu (T.S.); harrigan@hawaii.edu (R.H.); 2 National Institute of Environmental Health Sciences, National Institutes of Health, Durham, NC 27709, USA; Payel.sil@nih.gov; 3 Escuela de Medicina, Tecnologico de Monterrey, Monterrey, NL 64710, Mexico

**Keywords:** prediabetes, 5-ALA, diabetes, perceived stress scale, mood, coping ability

## Abstract

In 2010, approximately 79 million Americans had prediabetes and about 50 percent of those individuals were 65 years and older. The most effective diabetes prevention method in prediabetic adults is lifestyle modification. However, despite the benefits of lifestyle change, diabetes prevalence continues to increase. Maintaining a regular exercise routine and a healthy eating plan may be difficult because of the negative emotional barriers (i.e., stress, mood) that a prediabetic individual faces. This is particularly evident in older individuals when you combine that with decreases in mobility and geriatric syndromes. A potential treatment for these emotional barriers is a natural supplement called 5-aminolevulinic acid (5-ALA). In the current study, the group included 154 participants, both men and women, ranging between the ages of 41 to 71 years old. The study design was a double-blind, randomized parallel-group study. The Psychosocial Depressive Symptoms Questionnaire (PDS) and the Perceived Stress Scale (PSS) were used to examine the effect of two doses of 5-ALA (15 mg and 50 mg) on various components of mood (i.e., hopefulness, loneliness, and motivation) and coping ability. Using SAS software, an ordered logistic regression model was used to analyze the association between the dose groups (control, 15 mg, and 50 mg) and the responses to the two questionnaires, the PDS and PSS, used in this study. An integrative literature review, using the PubMed database, searched for studies on the relationship between 5-ALA administration and mood and coping ability. Our literature review resulted in zero published articles. Next, we found that the intake of 5-ALA was significantly associated with improved coping ability (*p* = 0.004) and improved self-perception of effort spent (*p* = 0.002). Finally, we found a significant dose-dependent relationship for the association of 5-ALA intake on measures of effort (*p* = 0.003), loneliness (*p* = 0.006), and coping ability (*p* = 0.003). The 50 mg dose was more effective than the 15 mg dose in improving these measures. In conclusion, after 12 weeks of taking 5-ALA, we found significant improvements in self-perception of effort spent, loneliness, and coping ability in prediabetic middle age and older adults. Improved mood and coping ability may allow prediabetic individuals to overcome the emotional obstacles preventing them from maintaining a healthy lifestyle and ultimately, help them to avoid the development of diabetes.

## 1. Introduction

In the current study, we evaluate whether 5-aminulevulinic acid (5-ALA) may improve various components of mood among study participants with prediabetes using two questionnaires entitled Psychosocial Depressive Symptoms Questionnaire and the Perceived Stress Scale (See Supplementary Materials). Prediabetes or intermediate hyperglycemia is a high-risk state for the development of diabetes [1]. According to an American Diabetes Association panel of experts, up to 70 percent of prediabetic individuals will ultimately develop diabetes [1]. Prediabetes is defined by glycemic variables (e.g., blood glucose concentrations) that are higher than normal limits, but lower than the diagnostic criteria for diabetes [1]. The most effective diabetes prevention method in prediabetic individuals is lifestyle modification [1]. This finding is supported by evidence showing a 40 to 70 percent relative-risk reduction for diabetes development [1]. Lifestyle modifications include reducing body weight and moderate daily exercise [2].

However, despite the benefits of lifestyle change, diabetes prevalence continues to increase [3]. In the U.S., diabetes affects 25.8 million Americans [4]. That is, more than 1 in 10 adults have diabetes and the prevalence of diabetes continues to rise in adults [3,4,5]. In fact, over the last 30 years the prevalence has increased by 176 percent [3]. This increase may be related to the many emotional health obstacles a prediabetic adult faces when it comes to maintaining an exercise routine and a healthy eating plan. Some examples of these emotional health obstacles are fatigue, poor sleep, mood swings, irritability, anxiety and depression [6,7]. Finding a solution to treat these emotional health issues may help prediabetic individuals maintain the healthy lifestyle modifications necessary to prevent the development of diabetes.

**Figure 1:**
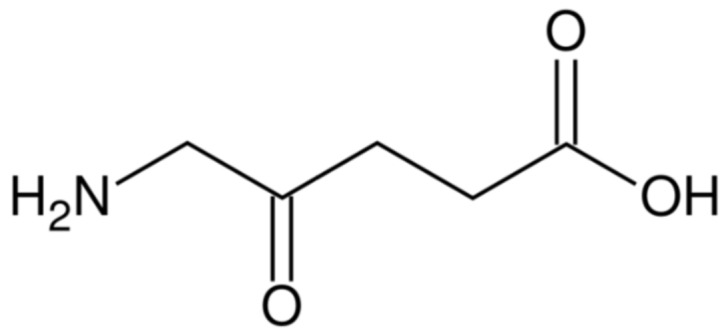
The chemical structure of 5-ALA.

A potential treatment for prediabetic emotional health issues, such as mood, is 5-ALA. This natural compound is a heme precursor in mammals and a chlorophyll precursor in plants (See Figure 1) [8,9]. Many common foods contain 5-ALA such as spinach, green peppers, tomatoes, bananas, and ground beef [10]. In previous publications, using the same study design and group of participants, previous authors reported that the administration of 5-ALA improves sleep [11] and reduces two hour post-oral glucose tolerance test (OGTT) glucose levels [12]. Specifically, Rodriguez and colleagues (2012) reported that among individuals taking 5-ALA for 12 weeks, two hour post–OGTT glucose levels declined significantly compared to those not taking the supplement (*p* = 0.02) [12]. The relationship was greater among those with glucose measurements greater than 140 mg/dL (*p* = 0.005 and *p* = 0.02 for the low and high dose groups, respectively) [12]. Similar trends were observed for Hemoglobin A1c but results were of borderline significance (*p* = 0.07) [12]. More recently, other studies have confirmed the beneficial effect of 5-ALA on glucose metabolism [13,14,15,16]. The health applications of 5-ALA, something that is currently not fully understood, is critical in designing the most effective wellness and diabetes prevention program.

In this study, our first aim is to perform an integrative literature review of the relationship between the administration of 5-ALA with mood and coping ability. The second aim is to determine the relationship between 15 mg and 50 mg 5-ALA and mood and coping ability using the Psychosocial Depressive Symptoms (PDS) and Perceived Stress Scale (PSS) Questionnaires. The final aim is to determine if a dose-dependent relationship exists between 5-ALA, mood, and coping ability.

## 2. Materials and Methods

### 2.1. Study Design

This research study uses previously collected data from the Prediabetes and Supplement Study (PASS). The PASS is a double-blinded randomized parallel-group comparison prospective study using participants residing on Oahu, Hawaii. Recruitment of study participants utilized IRB-approved local newspaper advertisements, flyers, television and radio advertising, referrals from local physicians, and local seminars and community programs. The study group includes 154 participants, both men and women, between the ages of 40 to 70 years old. All enrolled participants signed the IRB-approved informed consent. The selection of study participants was based on the strict inclusion and exclusion criteria provided in Table 1. For further details on study design, please see the paper by Rodriguez and colleagues [12].

### 2.2. Procedures

Potential participants were first screened and if eligible, consented at the initial visit. If eligible, participants were enrolled and asked to take a nutritional supplement (5-ALA) once a day for 90 days. The participants were randomly allocated to the following three study groups described in Table 2: control group (*n* = 51), 15 mg low dose of 5-ALA (*n* = 50), and 50 mg high dose of 5-ALA (*n* = 53). As described in Table 2, the control group was given a placebo capsule of identical size and color. The 5-ALA supplement used in this study contains the following components: (1) 5-ALA, (2) sodium ferrous citrate (SFC), and (3) corn starch as a filler [12]. For further information about the 5-ALA supplement used, please review the paper by Rodriguez and colleagues [12]. Table 3 describes the administration of the 5-ALA supplement. Participants were required to report for periodic physical exams the initial screening visit and every four weeks thereafter (Week 4, 8, 12, and 16). During these periodic check-ups, blood samples were collected. Blood samples were sent to the Diagnostic Laboratory Services of Hawaii for analysis.

### 2.3. Data Collection Tools

Table 4 and Table 5 describe the study procedures. Study participants were interviewed about diet, smoking status, and exercise or physical activity. The Prediabetes and Supplement Study (PASS) assessment tool, adapted from the Honolulu Heart Study [17], was used to assess lifestyle and physical activity. The Perceived Stress Scale (PSS) was used to measure stress [18]. A calibrated digital precision scale and a stadiometer were used to measure the participant’s weight and height, respectively. Body mass index (BMI) was calculated using weight (kg) divided by height (m^2^). A calibrated digital sphygmomanometer was used to measure blood pressure. The average of two measurements was used to estimate systolic and diastolic pressures. Finally, blood was collected at the initial visit and at the 12 week visit. The oral glucose tolerance test (OGTT) was also performed at the 12 week visit. For this test, participants fasted for 10 h prior to blood collection. The Diagnostic Laboratory Services of Hawaii performed the standardized blood analyses.

### 2.4. Questionnaires

The two questionnaires used in this study were the Psychological Depressive Symptoms (PDS) Questionnaire and the Perceived Stress Scale (PSS). These questionnaires are included in Supplementary Materials. The Psychological Depressive Symptoms questionnaire is a part of the Prediabetes and Supplement Study (PASS) assessment tool, adapted from the Honolulu Heart Study [17]. The Psychological Depressive Symptoms Questionnaire was designed to measure self-perception of mood. The Perceived Stress Scale, designed by Dr. Sheldon Cohen, is the most widely used psychological tool to measure self-perception of stress [18]. The questions in the PSS ask the respondent about his or her feelings during the last month regarding certain situations in one’s life [18]. For each situation, the PSS is designed to measure the degree to which a situation is considered stressful [18]. Furthermore, since the questions of both questionnaires are written in a general nature, they are relatively free of content specific to any sub-population group [18]. For both questionnaires, participants were asked about their thoughts and feelings during the last month towards each question. The answer choice options for both questionnaires were: “never,” “almost never,” “sometimes,” “fairly often,” and “very often.”

### 2.5. Literature Review Procedures

This literature review was focused on clinical trials, cross-sectional, case-control, and prospective cohort studies on the relationship between administrations of 5-ALA with mood and coping ability. A systematic search was conducted up to March 2018 using the PubMed database (MEDLINE, National Library of Medicine, Bethesda, MD, USA). To build a better query in PubMed, MeSH (Medical Subheadings) was utilized. MeSH is the National Library of Medicine controlled vocabulary thesaurus used for indexing articles for PubMed. In PubMed, the MeSH terms used were “5-ALA” or “5 5-aminolevulinic acid” along with other key words: “Prediabetes”, “Hyperglycemia”, “Diabetes”, “Mood”, “Coping”, “Emotional Health”, and “Psychological Health”. The search included both animal and human studies. The search was restricted to articles in English. All full-text studies were considered. This initial search resulted in zero articles. A further search was done using a list of relevant publications provided by SBI Pharmaceuticals Co., Ltd. This search resulted in zero articles. A final search using the above search terms was done using the Google Internet search engine and this also resulted in zero articles.

### 2.6. Statistical Analyses

Descriptive and inferential statistics were used to evaluate relationships between and among variables. The statistical programs utilized were SAS software, version 10 (SAS Institute Inc., Cary, NC, USA) and GraphPad Prism software, version 6 (GraphPad Software Inc., La Jolla, CA, USA). For the inferential tests, statistical significance was defined as *p* < 0.05. Using Prism software, a two-way ANOVA was performed to compare age and gender in the three groups. Using SAS software, an ordered logistic regression or proportional odds model was used to analyze the association between the dose groups (control, 15 mg, and 50 mg) and responses to the Psychosocial Depressive Symptoms (PDS) Questionnaire and Perceived Stress Scale (PSS). The appropriateness of the proportional odds model was tested using scored *p*-values.

## 3. Results

### 3.1. Participant Demographics

The study group includes 154 participants, both men and women, ranging between the ages of 41 to 71 years old. The participants were randomly allocated to the following three study groups described in Table 2: control group (*n* = 51), 15 mg low dose of 5-ALA (*n* = 50), and 50 mg high dose of 5-ALA (*n* = 53). The control group included 30 women and 21 men, the low dose group included 30 women and 20 men, and the high dose group included 33 women and 20 men. There were no significant differences in age and gender for any of the three groups (Group, *p* = 0.10; Gender, *p* = 0.59; Interaction, *p* = 0.76). Table 6 summarizes age and gender for each of the three groups. For further detail about study design and baseline characteristics of study participants please refer to the paper by Rodriguez and colleagues [12].

### 3.2. Group Comparisons

#### 3.2.1. Intake of 5-ALA and the Psychological Depressive Symptoms (PDS) Questionnaire

**Figure 2:**
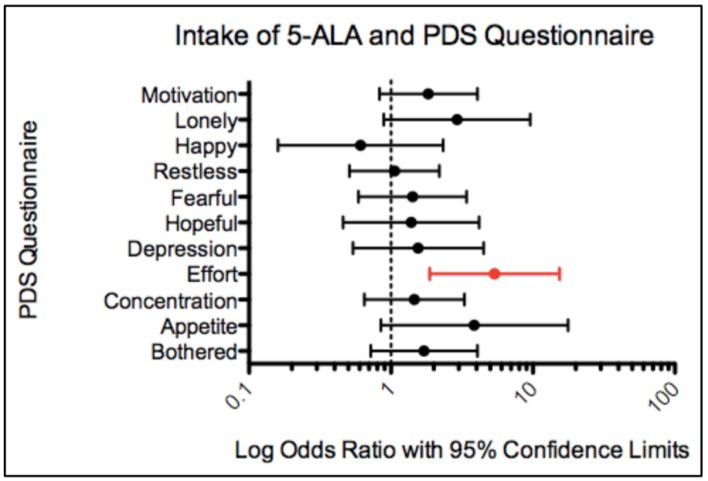
Intake of 5-ALA and Psychological Depressive Symptoms Questionnaire. Significant odds ratios are highlighted in red, *p* < 0.05.

In the analysis of the association of 5-ALA on responses to the PDS Questionnaire, only the outcome “effort” was found to be significant (*p* = 0.002). Table 7 and Figure 2 summarize the results of the effect on 5-ALA intake on each outcome variable from the PDS Questionnaire. The proportional odds model was appropriate for this analysis since all of the scored *p*-values were not significant.

#### 3.2.2. Intake of 5-ALA and the Perceived Stress Scale (PSS)

**Figure 3:**
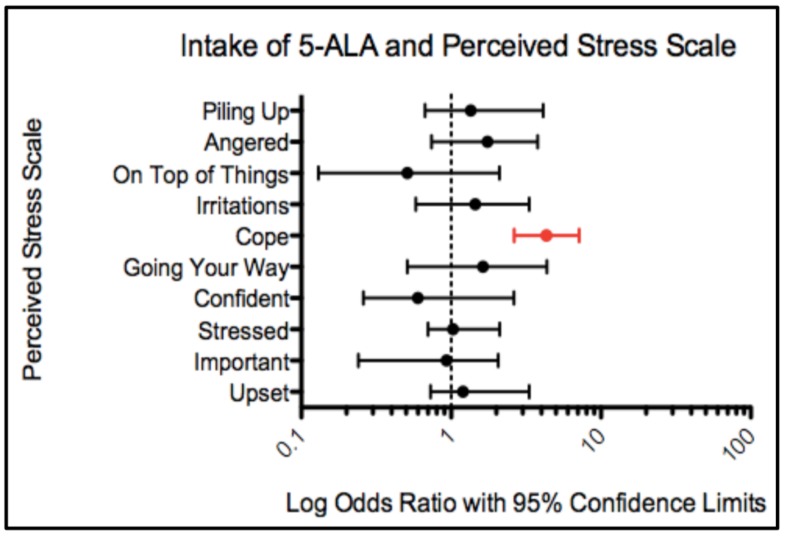
Intake of 5-ALA and the Perceived Stress Scale. Significant odds ratios are highlighted in red, *p* < 0.05.

In the analysis of the effect of 5-ALA on responses to the PSS Questionnaire, only the outcome “cope” was found to be significant (*p* = 0.004). Table 8 and Figure 3 summarize the results of the effect of 5-ALA intake on each outcome variable from the PSS Questionnaire. The proportional odds model was appropriate for this analysis since all of the scored *p*-values were not significant.

#### 3.2.3. Intake of 15 mg and 50 mg 5-ALA and the Psychological Depressive Symptoms (PDS) Questionnaire

**Figure 4:**
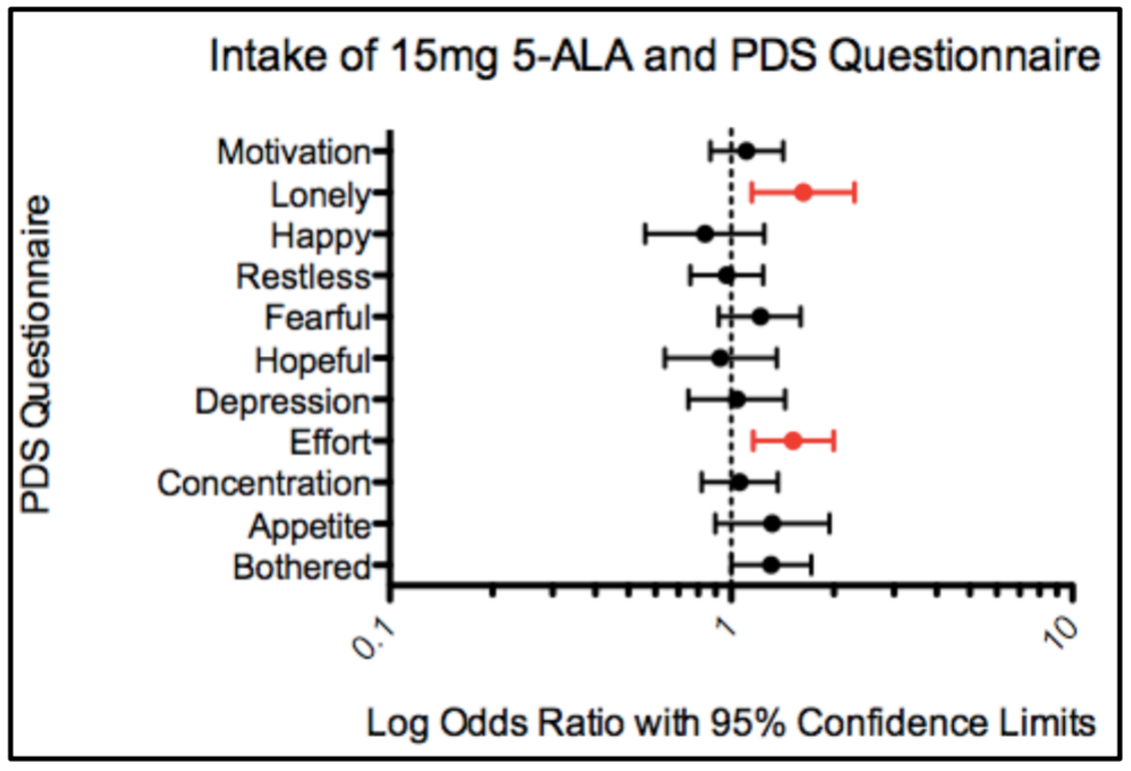
Intake of 15 mg 5-ALA and the Psychological Depressive Symptoms Questionnaire. Significant odds ratios are highlighted in red, *p* < 0.05.

**Figure 5:**
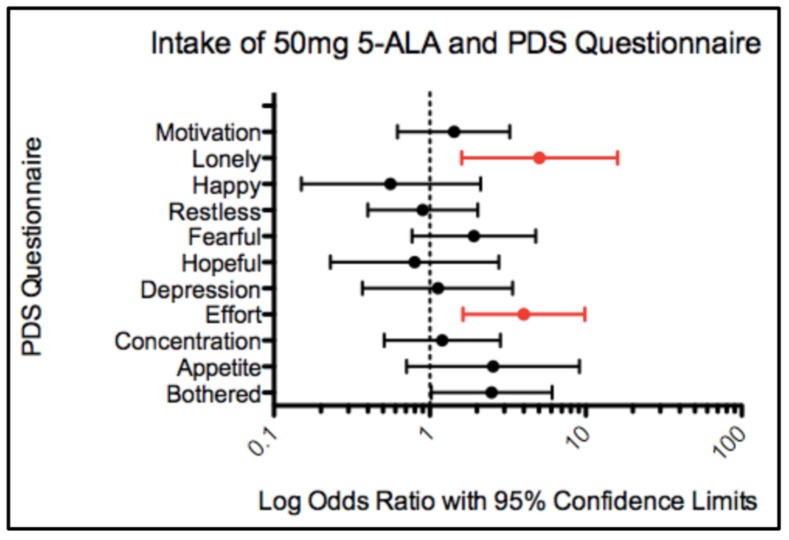
Intake of 50 mg 5-ALA and the Psychological Depressive Symptoms Questionnaire. Significant odds ratios are highlighted in red, *p* < 0.05.

In the analysis of the effect of 5-ALA (either 15 mg and 50 mg dose) on responses to the PDS Questionnaire, the outcomes “effort” and “lonely” were found to be significant (effort, *p* = 0.003; lonely, *p* = 0.006). Table 9 and Figure 4 and Figure 5 summarize the results of the effect for both 15 mg and 50 mg 5-ALA intake on each outcome variable from the PDS Questionnaire. The proportional odds model was appropriate for this analysis since all of the scored *p*-values were not significant.

#### 3.2.4. Intake of 15 mg and 50 mg 5-ALA and the Perceived Stress Scale (PSS)

**Figure 6:**
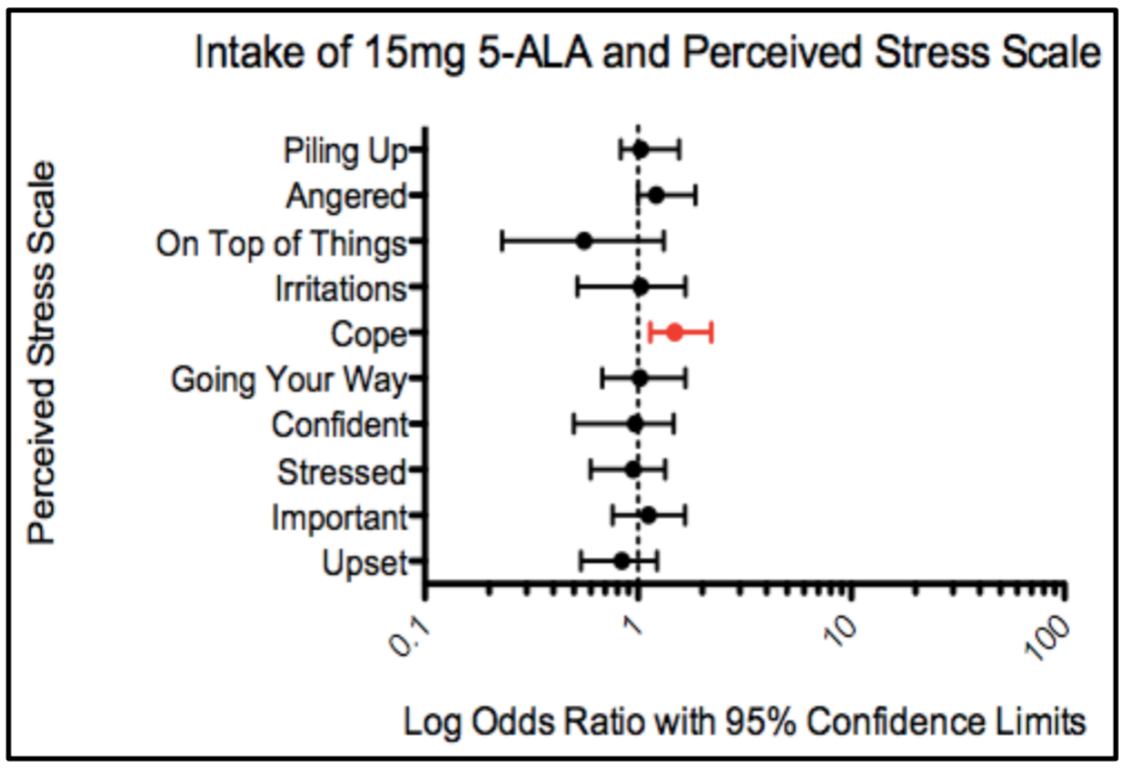
Intake of 15 mg 5-ALA and the Perceived Stress Scale. Significant odds ratios are highlighted in red, *p* < 0.05.

**Figure 7:**
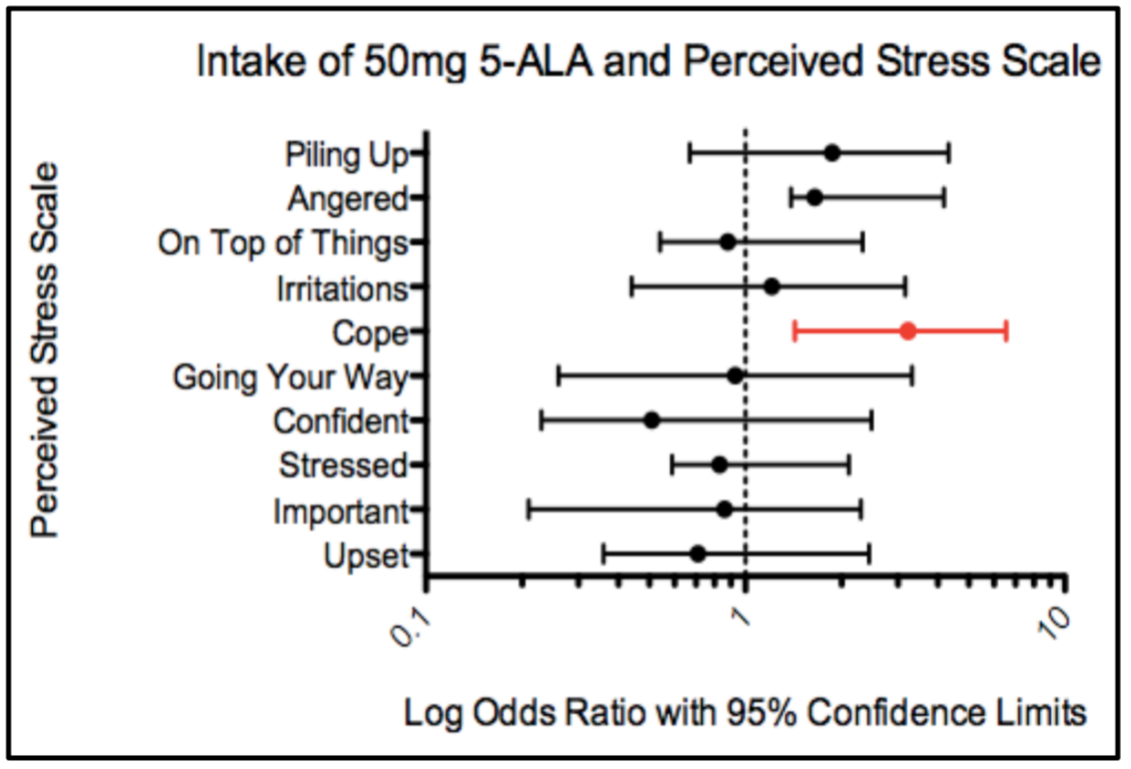
Intake of 50 mg 5-ALA and the Perceived Stress Scale. Significant odds ratios are highlighted in red, *p* < 0.05.

In the analysis of the effect of 5-ALA (either 15 mg and 50 mg dose) on responses to the PSS Questionnaire, only the outcome “cope” was found to be significant (cope, *p* = 0.004). Table 10 and Figure 6 and Figure 7 summarize the results of the effect for both 15 mg and 50 mg 5-ALA intake on each outcome variable from the PSS Questionnaire. The proportional odds model was appropriate for this analysis since all of the scored *p*-values were not significant.

## 4. Discussion

To summarize, the three specific aims of this study were met. First, we completed an integrative literature review that resulted in zero articles published on the relationship between the administration of 5-ALA and mood and coping ability. To the best of our knowledge, this is the first study to report the effect of 5-ALA intake on mood and coping ability in prediabetic middle aged and older adults. Second, utilizing the Psychological Depressive Symptoms (PDS) Questionnaire and the Perceived Stress Scale (PSS), the intake of 5-ALA was significantly associated with improved coping ability and improved self-perception of effort spent. Finally, a dose-dependent relationship was found for the effect of 5-ALA intake on measures of effort, loneliness, and coping ability. The 50 mg dose was more effective than the 15 mg dose in improving these measures. Overall, the intake of 5-ALA improved mood and coping ability in a population of prediabetic middle aged and older adults.

According to the Psychological Depressive Symptoms questionnaire, the intake of 5-ALA after 12 weeks improved self-perception of effort spent (See Supplementary Materials, PDS Question 4: “I felt that everything I did was an effort”). Compared to the participants’ answers at Week 1 (no 5-ALA), the intake of the 15 mg and 50 mg of 5-ALA significantly improved their perception of effort spent by 1.5 and 4.0 times, respectively. An improvement in self-perception of effort spent is important in helping prevent the development of diabetes. This is because while the most effective diabetes prevention strategy is lifestyle modification [1], for some individuals the task of maintaining healthy lifestyle changes may feel like an enormous effort or task.

The Psychological Depressive Symptoms questionnaire also revealed that the intake of 5-ALA significantly improved self-perception of loneliness (See Supplementary Materials, PDS Question 10: “I felt lonely”). Compared to the participants’ answers at Week 1 (no 5-ALA), 15 mg and 50 mg of 5-ALA significantly improved their perception of feeling alone by 1.6 and 5.1 times, respectively. Interestingly, it has been shown that loneliness depends on perception rather than social situation [19]. Often patients withdraw from family and friend support because they feel isolated or different from other people [20]. Some diabetic patients contribute the feeling of isolation with the responsibilities that come with their condition (i.e., diet restriction, checking blood glucose, insulin shots, etc.) [20]. Improving self-perception of loneliness is an important first step in preventing diabetes because prediabetic adults who have help from family, friends, and a community of similar patients are more likely to maintain a healthier lifestyle [21].

The Perceived Stress Scale (PSS) revealed that coping ability was improved in a group of prediabetic adults (See Supplementary Materials, PSS Question 6: “How often have you found that you could not cope with all the things that you had to do?”). Compared to the participants’ answers at Week 1 (no 5-ALA), 15 mg and 50 mg of 5-ALA after 12 weeks significantly improved coping ability by 1.5 and 3.2 times, respectively. Diabetes is a stressful, incurable disease that is associated with a higher incidence of depression and anxiety [22]. For prediabetic adults, the ability to cope with the threat of developing diabetes is important to minimize stress so they can focus on changing their lifestyle.

The mechanism for how 5-ALA improved mood and coping ability is beyond the scope of this project. However, there are a few possible mechanisms for improvement in mood and coping ability as a result of 5-ALA intake. In a previous study using a murine model, researchers found that regular administration of 5-ALA raised brain serotonin levels [23]. Another study also suggested that 5-ALA might improve mood through its influence on neuroactive substances such as tryptophan, serotonin and melatonin [10]. Researchers hypothesized that an increase in serotonin levels may correspond with improvements in mood, calmness, irritability, and coping abilities [23]. In addition, using the same study design and cohort as the current study, Perez and colleagues (2013) reported an improvement in sleep as a result of 5-ALA intake [10]. The authors suggest that improvement in sleep as a result of 5-ALA intake may be related to a boost in cellular metabolism [10]. It is well accepted that sleep and mood are closely linked [24]. Poor sleep is associated with irritability and stress, while adequate sleep is associated with enhanced well-being [24]. Improvement in sleep in prediabetic adults may increase the energy needed to cope with the possibility of being diagnosed with diabetes. Also, improved sleep may help prediabetic adults feel less alone. A previous study found that people who feel lonely experience more sleep disruptions compared to those who do not feel lonely [19].

In conclusion, we report that after 12 weeks of taking 5-ALA, self-perception of effort spent, loneliness, and coping ability were improved in a group of prediabetic middle aged and older adults. Improved mood and coping ability may allow prediabetic individuals to overcome the emotional obstacles preventing them from maintaining a healthy lifestyle and ultimately, help them stop the development of diabetes.

## 5. Conclusions

In conclusion, we report that after 12 weeks of taking 5-ALA, self-perception of effort spent, loneliness, and coping ability were improved in a group of prediabetic middle aged and older adults. Improved mood and coping ability may allow prediabetic individuals to overcome the emotional obstacles preventing them from maintaining a healthy lifestyle and ultimately, help them stop the development of diabetes.

## Figures and Tables

**Table 1:** Inclusion and Exclusion Criteria.

**Inclusion Criteria**
(1) Healthy adults living in Oahu, Hawaii or able to attend five on-site one hour appointments over a 12 week period
(2) Body weight between 110 and 250 pounds
(3) Normal CBC and ferritin laboratory analysis done at initial screening
(4) Hemoglobin A1c level range from 5.8% to 7.0%
(5) Fasting blood sugar level range from 110 to 125 (Impaired Glucose Tolerance range from 140 to 199 at 2 h post load)
**Exclusion Criteria**
(1) History of hepatitis, porphyria, hemochromatosis, and iron sensitivity
(2) Active liver disease
(3) Laboratory analysis with elevated ferritin levels above 125% normal levels obtained at initial screening visit
(4) Current participation in another clinical research study
(5) For women, pregnancy and breastfeeding
(6) Body Mass Index value of more than 30 and a body weight of <100 or >250 pounds
(7) Medical prescription that affects blood sugar level

**Table 2:** Group and Supplement Description.

Supplement Content	Control Group (mg)	Low Dose Group (mg)	High Dose Group (mg)
5-ALA Phosphate	0 mg	15 mg	50 mg
Sodium Ferrous Citrate (SFCi)	0 mg	17.2 mg (1.82 mg as Iron)	57.4 mg (6.08 mg as Iron)
Other	Alpha starch, silicon dioxide	Alpha starch, silicon dioxide	Alpha starch, silicon dioxide

**Table 3:** Supplement Schedule.

Visit Number	Visit Description	Timeline
1	Screening	Screening
2	Intervention period	Day-0
3	Intervention period	Week-4
4	Intervention period	Week-8
5	Intervention period	Week-12
6	Follow-up	Week-16

**Table 4:** Study Procedures by Assessment.

Assessment Type	Assessment Description
Lifestyle-related questionnaires	Self-reported sleep history, food consumption, exercise and physical activitiy, nutrient intake, drug history including prescription, illicit, alcohol, and nicotine
Clinical examination	Medical history, body weight, height, BMI, systolic/diastolic blood pressure, heart rate, waist circumference, Oral Glucose Tolerance Test (OGTT)
Laboratory tests	Hepatic function (ALT, AST, LDH, γ-GTP, T-Bill, Alb, TP, urine bilirubin), Renal Function (BUN, creatinine, uric acid, urinary protein, occult blood), Inflammation/Infection (WBC count, fibrinogen), Hyperlipidemia (total cholesterol, HDL-chr, LDL-chr, triglyceride), Anemia (RBC count, hemoglobin level, hematocrit, blood platelet count, serum iron level, ferritin, transferrin, UIBC, TIBC, MCH, MCV, MCHC), Glucose metabolism (blood glucose, urinary glucose, urinary pH, HbA1c), Electrolyte metabolism (Na, K, Cl)
Dietary inquiry	Study diary, self-reported symptoms, use of dietary supplements
Psychiatric questionnaires	Perceived Stress Scale, Psychosocial Depressive Symptoms Questionnaire

**Table 5:** Study Procedures by Study Task.

Study Task	Visit 1	Visit 2	Visit 3	Visit 4	Visit 5	Visit 6
Lifestyle, PDS, PSS, & Food Questionnaires		X			X	
Clinical Exam	X	X	X	X	X	X
Lab Tests	X	X	X	X	X	X
Oral glucose tolerance test (OGTT)		X			X	
Intake of supplements (5-ALA or placebo)		X	X	X	X	X
Study Diary	X	X	X	X	X	X

**Table 6:** Age and Gender by Group with Means and Standard Errors.

Group	Men			Women		
	Mean	SEM	N	Mean	SEM	N
High Dose Group	58.0	2.1	20	57.3	1.3	33
Low Dose Group	57.3	2.0	20	57.7	1.4	30
Control	61.5	1.4	21	59.6	1.3	30

A Two-way ANOVA resulted in no significant differences: Group, *p* = 0.10; Gender, *p* = 0.59; Interaction, *p* = 0.76.

**Table 7:** Intake of 5-ALA and the Psychosocial Depressive Symptoms Questionnaire.

Outcome	Odd’s Ratio	Lower CL *	Upper CL *	*p* Value	Scored *p* Value	Predictor
Bothered	1.71	0.72	4.06	0.23	0.54	ALA
Poor Appetite	3.86	0.85	17.67	0.08	0.90	ALA
Poor Concentration	1.46	0.65	3.29	0.36	0.09	ALA
Effort	5.36	1.87	15.38	0.002	0.61	ALA
Depressed	1.55	0.54	4.49	0.42	0.89	ALA
Hopeful Future	1.39	0.46	4.17	0.56	0.58	ALA
Fearful	1.42	0.59	3.41	0.44	0.41	ALA
Restless Sleep	1.06	0.51	2.19	0.88	0.16	ALA
Happy	0.61	0.16	2.33	0.47	0.27	ALA
Lonely	2.92	0.89	9.58	0.08	0.41	ALA
Unmotivated	1.83	0.83	4.06	0.14	0.69	ALA

* 95% confidence interval.

**Table 8:** Intake of 5-ALA and the Perceived Stress Scale.

Outcome	Odd’s Ratio	Lower CL *	Upper CL *	*p* Value	Scored *p* Value	Predictor
Upset	1.20	0.73	3.32	0.26	0.44	ALA
Important	0.93	0.24	2.06	0.55	0.58	ALA
Stressed	1.03	0.70	2.11	0.67	0.24	ALA
Confident	0.60	0.26	2.63	0.54	0.59	ALA
Going Your Way	1.63	0.51	4.35	0.44	0.89	ALA
Cope	4.32	2.64	7.16	0.004	0.50	ALA
Irritation	1.45	0.58	3.32	0.63	0.66	ALA
On Top of Things	0.51	0.13	2.10	0.57	0.45	ALA
Angered	1.75	0.74	3.78	0.16	0.56	ALA
Piling Up	1.35	0.67	4.12	0.51	0.71	ALA

* 95% confidence interval.

**Table 9:** Intake of 15 mg and 50 mg 5-ALA and the Psychological Depressive Symptoms Questionnaire.

Outcome	Odd’s Ratio	Lower CL *	Upper CL *	*p* Value	Scored *p* Value	Predictor
Bothered-Low Dose	1.31	1.72	1.00	0.05	0.54	ALA 15 mg
Bothered-High Dose	2.49	6.08	1.02	0.05	0.54	ALA 50 mg
Poor Appetite-Low Dose	1.32	1.94	0.90	0.15	0.90	ALA 15 mg
Poor Appetite-High Dose	2.55	9.11	0.71	0.15	0.90	ALA 50 mg
Poor Concentration-Low Dose	1.06	1.37	0.82	0.68	0.09	ALA 15 mg
Poor Concentration-High Dose	1.20	2.84	0.51	0.68	0.09	ALA 50 mg
Effort-Low Dose	1.52	2.00	1.16	0.003	0.61	ALA 15 mg
Effort-High Dose	4.01	9.88	1.63	0.003	0.61	ALA 50 mg
Depressed-Low Dose	1.04	1.44	0.75	0.83	0.89	ALA 15 mg
Depressed-High Dose	1.13	3.39	0.37	0.83	0.89	ALA 50 mg
Hopeful Future-Low Dose	0.93	1.36	0.64	0.72	0.58	ALA 15 mg
Hopeful Future-High Dose	0.80	2.77	0.23	0.72	0.58	ALA 50 mg
Fearful-Low Dose	1.22	1.60	0.92	0.16	0.41	ALA 15 mg
Fearful-High Dose	1.92	4.78	0.77	0.16	0.41	ALA 50 mg
Restless Sleep-Low Dose	0.97	1.24	0.76	0.80	0.16	ALA 15 mg
Restless Sleep-High Dose	0.90	2.03	0.40	0.80	0.16	ALA 50 mg
Happy-Low Dose	0.84	1.25	0.56	0.39	0.27	ALA 15 mg
Happy-High Dose	0.56	2.12	0.15	0.39	0.27	ALA 50 mg
Lonely-Low Dose	1.63	2.30	1.15	0.006	0.41	ALA 15 mg
Lonely-High Dose	5.05	16.00	1.60	0.006	0.41	ALA 50 mg
Unmotivated-Low Dose	1.11	1.42	0.87	0.40	0.69	ALA 15 mg
Unmotivated-High Dose	1.43	3.25	0.62	0.40	0.69	ALA 50 mg

* 95% confidence interval.

**Table 10:** Intake of 15 mg and 50 mg 5-ALA and the Perceived Stress Scale.

Outcome	Odd’s Ratio	Lower CL *	Upper CL *	*p* Value	Scored *p* Value	Predictor
Upset-Low Dose	0.84	0.54	1.23	0.45	0.44	ALA 15 mg
Upset-High Dose	0.71	0.36	2.44	0.45	0.44	ALA 50 mg
Important-Low Dose	1.12	0.76	1.66	0.37	0.58	ALA 15 mg
Important-High Dose	0.86	0.21	2.30	0.37	0.58	ALA 50 mg
Stressed-Low Dose	0.95	0.60	1.34	0.81	0.24	ALA 15 mg
Stressed-High Dose	0.83	0.59	2.11	0.81	0.24	ALA 50 mg
Confident-Low Dose	0.97	0.50	1.47	0.56	0.59	ALA 15 mg
Confident-High Dose	0.51	0.23	2.48	0.56	0.59	ALA 50 mg
Going Your Way-Low Dose	1.02	0.68	1.67	0.93	0.89	ALA 15 mg
Going Your Way-High Dose	0.93	0.26	3.32	0.93	0.89	ALA 50 mg
Cope-Low Dose	1.49	1.14	2.21	0.003	0.50	ALA 15 mg
Cope-High Dose	3.32	1.43	6.54	0.003	0.50	ALA 50 mg
Irritation-Low Dose	1.03	0.52	1.67	0.78	0.66	ALA 15 mg
Irritation-High Dose	1.21	0.44	3.17	0.78	0.66	ALA 50 mg
On Top of Things-Low Dose	0.56	0.23	2.33	0.50	0.45	ALA 15 mg
On Top of Things-High Dose	0.88	0.54	1.32	0.50	0.45	ALA 50 mg
Angered-Low Dose	1.22	1.00	1.86	0.65	0.56	ALA 15 mg
Angered-High Dose	1.65	1.39	4.18	0.65	0.56	ALA 50 mg
Piling Up-Low Dose	1.03	0.83	1.56	0.23	0.71	ALA 15 mg
Piling Up-High Dose	1.87	0.67	4.33	0.23	0.71	ALA 50 mg

* 95% confidence interval.

## Supplementary Materials

The following are available at http://www.mdpi.com/2308-3417/3/2/17/s1. S1: Perceived Stress Scale, S2: Psychological Depressive Symptoms Questionnaire.

## Abbreviations

The following abbreviations are used in this manuscript:

5-ALA	5-Aminolevulinic Acid
ADA	American Diabetes Association
ANOVA	Analysis of Variance
BMI	Body Mass Index
CBC	Complete Blood Count Test
IRB	Institutional Review Board
OGTT	Oral Glucose Tolerance Test
PASS	Prediabetes and Supplement Study
PDS	Psychosocial Depressive Symptoms Questionnaire
PSS	Perceived Stress Scale
SAS	Statistical Analysis System
SBI	Strategic Business Innovations
