# Supplementary material for: The Relationship of 5-Aminolevulinic Acid on Mood and Coping Ability in Prediabetic Middle Aged and Older Adults
Source: Geriatrics (Basel). 2018 Apr 4;3(2):17. doi: 10.3390/geriatrics3020017 (PMC5976501; doi:10.3390/geriatrics3020017)
Supplement: Supplementary file 1 [file geriatrics-03-00017-s001.zip › Perceived Stress Scale.pdf]

Instructions: The following questions ask you about your feelings and thoughts during the last month. In each case, please indicate with a check how often you felt or thought a certain way.

1. In the last month, how often have you been upset because of something that happened unexpectedly?

___0=never ___1=almost never ___ 2=sometimes ___3=fairly often ___4=very often

2. In the last month, how often have you felt that you were unable to control the important things in your life?

___0=never ___1=almost never ___ 2=sometimes ___3=fairly often ___4=very often

3. In the last month, how often have you felt nervous or "stressed"?

___0=never ___1=almost never ___ 2=sometimes ___3=fairly often ___4=very often

4. In the last month, how often have you felt confident about your ability to handle your personal problems?

___0=never ___1=almost never ___ 2=sometimes ___3=fairly often ___4=very often

5. In the last month, how often have you felt that thinks were going your way?

___0=never ___1=almost never ___ 2=sometimes ___3=fairly often ___4=very often

6. In the last month, how often have you found that you could not cope with all the things that you had to do?

___0=never ___1=almost never ___ 2=sometimes ___3=fairly often ___4=very often

7. In the last month, how often have you been able to control irritations in your life?

___0=never ___1=almost never ___ 2=sometimes ___3=fairly often ___4=very often

8. In the last month, how often have you felt that you were on top of things?

___0=never ___1=almost never ___ 2=sometimes ___3=fairly often ___4=very often

9. In the last month, how often have you been angered because of things that were outside of your control?

___0=never ___1=almost never ___ 2=sometimes ___3=fairly often ___4=very often

10. In the last month, how often have you felt difficulties were piling up so high that you could not overcome them?

___0=never ___1=almost never ___ 2=sometimes ___3=fairly often ___4=very often
