# Supplementary material for: The Relationship of 5-Aminolevulinic Acid on Mood and Coping Ability in Prediabetic Middle Aged and Older Adults
Source: Geriatrics (Basel). 2018 Apr 4;3(2):17. doi: 10.3390/geriatrics3020017 (PMC5976501; doi:10.3390/geriatrics3020017)
Supplement: Supplementary file 1 [file geriatrics-03-00017-s001.zip › Pschological Depressive Symptoms Questionnaire.pdf]

PSYCHOSOCIAL - DEPRESSIVE SYMPTOMS

Participant is unable or unwilling to complete this section.	No	Yes
	0	1

If "Yes", specify reasons and skip this page.

Below is a list of the ways you might have felt or behaved.
Please indicate how often you have felt this way during the past week.

Would you say in the last week	Rarely or none of time (less than 1 day)	Some or little of the time (1 - 2 days)	Occasionally or a moderate amount of the time (3 - 4 days)	Most of the time	Don't know or refused
1. I was bothered by things that usually don't bother me.	0	1	2	3	9
2. I did not feel like eating, my appetite was poor.	0	1	2	3	9
3. I had trouble keeping my mind on what I was doing.	0	1	2	3	9
4. I felt that everything I did was an effort.	0	1	2	3	9
5. I felt depressed	0	1	2	3	9
6. I felt hopeful about the future.	0	1	2	3	9
7. I felt fearful.	0	1	2	3	9
8. My sleep was restless.	0	1	2	3	9
9. I was happy.	0	1	2	3	9
10. I felt lonely.	0	1	2	3	9
11. I could not get going.	0	1	2	3	9
